# Engineering Auxetic Cylinders and Intestine to Improve Longitudinal Intestinal Lengthening and Tailoring Procedure

**DOI:** 10.3390/bioengineering9110658

**Published:** 2022-11-06

**Authors:** Luca Valentini, Irene Chiesa, Carmelo De Maria, Sara Ugolini, Yary Volpe, Elisa Mussi, Lucia Pappalardo, Riccardo Coletta, Antonino Morabito

**Affiliations:** 1 Dipartimento di Ingegneria Civile e Ambientale, Università di Perugia, UdR INSTM, 05100 Terni, Italy; 2 Department of Ingegneria dell’Informazione and Research Center E. Piaggio, University of Pisa, 56124 Pisa, Italy; 3 Department of Cardiothoracic Surgery, Wythenshawe Hospital, Manchester University NHS Foundation Trust, Manchester M13 9WL, UK; 4 Dipartimento di Ingegneria Industriale, University of Florence, 50139 Florence, Italy; 5 Istituto Nazionale di Geofisica e Vulcanologia, Osservatorio Vesuviano, 80124 Napoli, Italy; 6 Department of Paediatric Surgery, Meyer Children’s Hospital, 50139 Florence, Italy; 7 School of Health and Society, University of Salford, Salford M5 4NT, UK

**Keywords:** short bowel syndrome, intestinal failure, reconstructive surgical procedures, mechanical modeling, auxetic materials, graphene

## Abstract

Auxetic materials can be exploited for coupling different types of tissues. Herein, we designed a material where the microorganism metabolic activity yields the formation of buckled/collapsed bubbles within gelling silicone cylinders thus providing auxetic properties. The finite element model of such hollow auxetic cylinders demonstrated the tubular structure to promote worm-like peristalsis. In this scenario, the described hybrid auxetic structures may be applied to the longitudinal intestinal lengthening and tailoring procedure to promote enteral autonomy in short bowel syndrome. The presented material and analytical design synergistic approach offer a pioneering step for the clinical translation of hybrid auxetic materials.

## 1. Introduction

When considering isotropic linear elastic materials, an intuitive thought is that they shrink/expand laterally when stretched/compressed axially (showing a positive Poisson’s ratio) [1,2,3]. Current research on polymer composites reports most of them shrinking laterally like a rubber band when stretched, meaning that their Poisson ratios are positive [4,5,6]. Likewise, most materials become thinner in a width-wise direction when stretched along their length. However, even if most materials (including foams) exhibit a positive Poisson’s ratio, theoretically, negative Poisson’s ratios (corresponding to an auxetic behavior) are permissible [7,8,9,10]. We previously reported [11] that the combined fermentation of natural microorganisms with nanostructured carbon materials (i.e., carbon nanotube (CNTs) and graphene nanoplatelets (GNPs)) results in an extreme auxetic deformation behavior. The combination of auxetic and non-auxetic materials may open new opportunities in the biomedical field by shape-morphing, creating unique hybrid structures and potentially leading to new clinical applications. Peristalsis, for example, is a progressive movement that shifts the chyme from the lumen of one intestinal segment to the downstream segment [12,13]. This action is generated by a sequential contraction–relaxation pattern caused by longitudinal and circular muscles with the peculiarity that the outer muscle layer (longitudinal) contracts and shortens, whereas in contrast, the inner layer (circular) relaxes and widens. Such worm-like peristalsis under simple uniaxial actuation could be simulated by the combination of auxetic and non-auxetic phases. Aiming to understand the clinical feasibility of this new bioengineering technology, we decided to mimic this material by mimicking the Longitudinal Intestinal Lengthening and Tailoring (LILT) procedure. LILT is one of the most performed procedures to treat Short Bowel Syndrome (SBS). Its goal is to promote enteral nutritional autonomy (and weaning off parenteral nutrition) by lengthening the viable bowel (autologous gastrointestinal reconstruction) [14,15,16,17]. LILT clinical benefits derived from an increased length and reduced lumen caliber, which limits the flow stasis and subsequent bacterial overgrowth, and maximizes the absorption of nutrients and fluids [17,18,19]. Unfortunately, the indications for LILT exclude unfavourable anatomy such as lack of bowel dilatation or damaged mesentery, which make the procedure unfeasible [20]. The latter is the rationale behind the need for synthetic substitutes to mimic the peristaltic movements. In this study, auxetic cylinders were designed starting from the observation that when a carbon dioxide (CO_2_) bubble is released by yeast fermentation, it deforms a gelling medium by contact [11]. In fact, the formation of porous composites thanks to yeast fermentation has important benefits, such as rapid pore formation, the utilization of sustainable inexpensive agents and the self-assembling of nanoparticles. In particular, floating GNPs/CNTs are captured by the rising CO_2_ bubbles and then transferred to the gelling polymer. Thus, nanomaterials are expected to self-assemble at the polymer/CO_2_ interface to form a new film that, due to the high interfacial tension of the two immiscible liquid phases entrapping the particles, increases the interfacial energy and minimizes the free energy of the system.

To this aim, Silicon Rubber (SR) was chosen as the gelling material due to its comprehensive utilization in various clinical applications, including tissue implants [21]. A Finite Element (FE) model was then developed, combining auxetic (i.e., SR cylinders) and non-auxetic materials (i.e., intestine) to evaluate the behavior of such metastructures.

The auxetic tubes interposition? between intestinal segments has shown to aid the peristaltic movement by reducing the pressure drop and, thus, the stagnation of the intestinal bolus. These shape-morphing characteristics are discussed to support the adoption of metamaterials to widen LILT indications to difficult cases with unfeasible anatomy.

## 2. Materials and Methods

### 2.1. Fabrication of Auxetic Cylinders

GNPs were supplied by NANESA (G3Nan, average thickness, 9 nm ≈ 25 layers, bulk density, 0.018–0.023 g cm^−3^, average lateral particle size, 15 µm). CNTs (CNTs, NC 7000) were purchased from Nanocyl (bulk density, 0.066 g cm^−3^, average diameter, 9.5 nm, average length, 1.5 µm). An *S. cerevisiae*-based commercial beer yeast extract was used as a medium for fermentation. Yeast fermentation was employed to introduce bubbles into the silicone rubber. A crystal liquid rubber (CRISTAL RUBBER purchased from PROCHIMA, density, 1.04 g cm^−3^) was used for casting with a cold cure by polyaddition. Before using the rubber, 10 wt% of PT-CURE catalyst (purchased from PROCHIMA, density, 1.04 g cm^−3^) was added. GNPs and CNTs were dispersed in three different amounts (i.e., 1 wt% of GNPs, 1 wt% of CNTs and 0.5 wt%/0.5 wt% CNTs/GNPs) in the liquid silicone rubber through a magnetic stirrer (500 rpm for 3 h) to facilitate their dispersion. After that, yeast (0.1 weight ratio concerning the liquid silicone rubber) and sugar (i.e., sucrose, 0.04 weight ratio concerning the liquid silicone rubber), previously dispersed in 2 mL of water and heated at 50 °C to start the fermentation, were added to the silicone mixture. The silicone mixture containing the yeast was heated at 50 °C, and then the catalyst was added. Once the fermentation process stopped, the polyaddition reaction was completed for 24 h at room temperature in a cylindrical mold (10 mm in diameter and 20 mm in height) consisting of two parts sealed by screws and silicone. The cylinder’s radius-to-height ratio (R/H) was fixed to R/H = 0.25.

### 2.2. Characterization

To simulate the human intestine’s mechanical properties, a porcine intestine fragment was tested with a tensile testing machine (Lloyd Instr. LR30K, West Sussex UK). Rectangular samples (1.5 cm × 3 cm × 100 μm) were stretched with a strain rate of 5 mm·min^−1^ using a 50 N load cell. Two cylindrical metal plates performed the compression test of the cylinders at a compression rate of 0.1 mm/s. The Poisson modulus was calculated by analyzing digital images through VIC 3D software. The microstructure of the samples was investigated by X-ray microtomography (μCT) using a Carl Zeiss Xradia Versa-410 3D X-ray microscope. In this study, cylinders of a diameter of 10 mm were scanned over a 360° rotation using 1601 projections, 60 KV voltage, and 5 W power. The resulting nominal voxel (volumetric pixel) size was 18 μm (the optical magnification used was 0.4×). The attenuation data were reconstructed through the filtered back-projection algorithm using XRM Reconstructor Xradia proprietary software, producing a stack of 967 cross-sectional, grey-scale digital images. Volume rendering and vesicularity, bubbles number and volume were obtained by processing the 3D tomographic images using Dragonfly Pro ORS software. The deviation from a spherical shape (i.e., anisotropy) versus elongation was measured on the pores by the software Avizo8.

### 2.3. Finite Element Modelling

**Figure 1:**
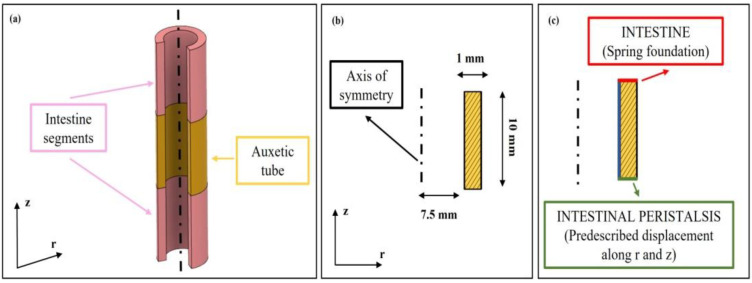
FE models of the silicone tube with different Poisson coefficients. (**a**) 3D representation of the modeled tube: the auxetic tube is interposed between two intestinal porcine segments. In the software, the tube was included as domain, whereas the property and movements of the intestines were included via boundary conditions. (**b**) Geometrical properties of the modelled silicone tube. (**c**) The green and red lines represent the imposed boundary conditions, whereas the blue line shows the boundary at which the quantities of interests were evaluated.

An FE model was implemented on Comsol Multiphysics (Comsol Inc., version 5.3, Stockholm, Sweden) to analyze effects on the peristaltic movement following the addition of the auxetic silicone tubes between sections of the intestine walls. The solid mechanics application mode in static conditions was used. A 2D axisymmetric model was designed in the software, comprising a single domain that models the silicone tube (Figure 1a). The geometrical dimensions are reported in Figure 1b. A linear elastic material model was used, whose subdomains settings are documented in Table 1. A parametric study was implemented varying the Poisson coefficient of the silicone tube (Table 1), including auxetic and non auxetic tubes. To reproduce the peristaltic movements of the intestine, a prescribed displacement of −3 mm along r and z was introduced as boundary condition (green line, Figure 1c), thus modelling the intestinal contraction where the silicone tube was sealed. Moreover, on the opposite boundary (red line, Figure 1c), a spring foundation was applied to constrain the structure, defined with a Young’s Modulus of 0.1 MPa, a Poisson ratio of 0.49 and a thickness of 10 mm, that mimicked the intestine wall. The displacements along r and z along the internal wall of the silicone tube (blue line, Figure 1c) were evaluated for each value of the Poisson coefficient. The silicone tube’s final inner diameter and length were obtained for each Poisson coefficient. Finally, the ratio between the tube’s final length and its final diameter was evaluated for each Poisson coefficient.

### 2.4. Mathematical Analysis of the LILT Procedure

**Figure 2:**
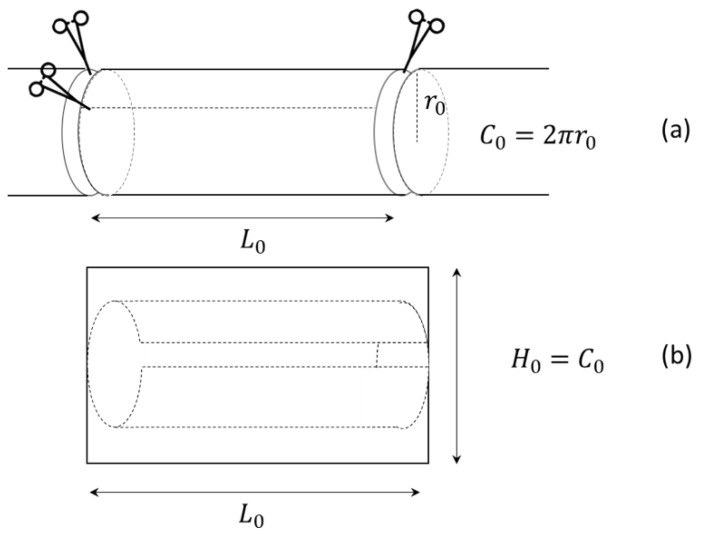
LILT procedure. (**a**) Removal of an intestine part of length $L_{o}$. (**b**) Developed cylinder.

To understand LILT morphological and physiological changes, we analyzed the primary bowel dimensional changes by considering a simplified geometrical model of the elongated segment. At first, when performing a LILT, the original bowel is reshaped into an open cylinder (Figure 2a) with a given length (*L*_o_) and radius (*r*_o_). Secondly, the length is increased at the expense of the circumference. To describe the process, the original surface area (*A*_o_) of the cylinder (bowel before surgery), its volume (*V*_o_) and its rim (*C*_o_) of the end cap are given by:

(1) $$A_{o}=2\pi r_{o}L_{o},\;V_{o}=\pi r_{o}^{2}L_{o},\;C_{o}=2\pi r_{o}.$$

As in the LILT procedure, when the cylinder is opened by a longitudinal cut, an approximate rectangle of height equal to $H_{o}=C_{o}$ and length $L_{o}$ is obtained (Figure 2).

**Figure 3:**
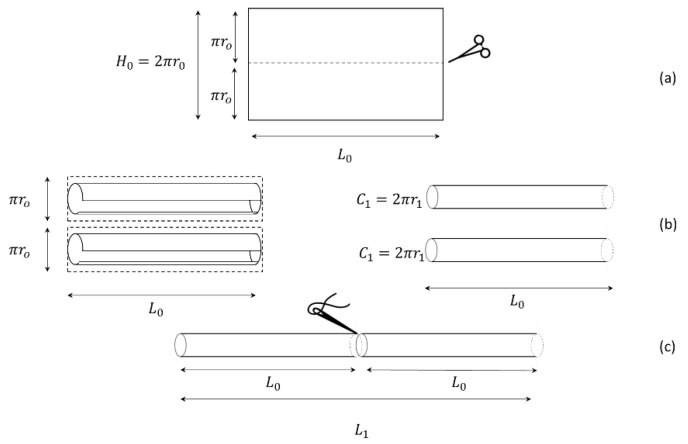
LILT procedure. (**a**) Longitudinal cut of the developed bowel in two equal rectangles. (**b**) Rolling of the two rectangles bowel around the long side. (**c**) Anatomical geometry of the new bowel cylinders.

The rectangular bowel was then cut in half, giving two equal rectangles (height $\pi r_{o}$ and length $L_{o}$), creating two new cylinders (hemi-loops), as shown in Figure 3.

The resulting two cylinders of length $L_{o}$ were then sutured, as shown in Figure 3c. This provided a bowel of the lengthened $L_{1}$, where:

(2) $$L_{1\;}\;=2L_{0}$$

and circumference $C_{1}=\pi r_{0}$, as shown in Figure 2. This resulted in a halved radius of the final bowel loop, as shown below:

(3) $$C_{1}=2\pi r_{1}=\pi r_{0}.$$

which gives

(4) $$r_{1}=\frac{r_{o}}{2}.$$

Therefore, by substituting $r_{0}$ and $L_{0}$, the new area of the two lengthened cylinders was:

(5) $$A_{1}=2\pi r_{1}L_{1}=2\pi \frac{r_{o}}{2}2L_{o}=2\pi r_{o}L_{o}={\;A}_{0}$$

which was equal to the area of the original cylinder. In contrast, by substituting $r_{0}$ and $L_{0}$, the new volume of the two lengthened cylinders was:

(6) $$V_{1}=\pi r_{1}^{2}L_{1}=\pi {\left(\frac{r_{0}}{2}\right)}^{2}2L_{o}=\frac{\pi r_{0}^{2}L_{o}}{2}=\frac{V_{0}}{2}$$

## 3. Results and Discussion

**Figure 4:**
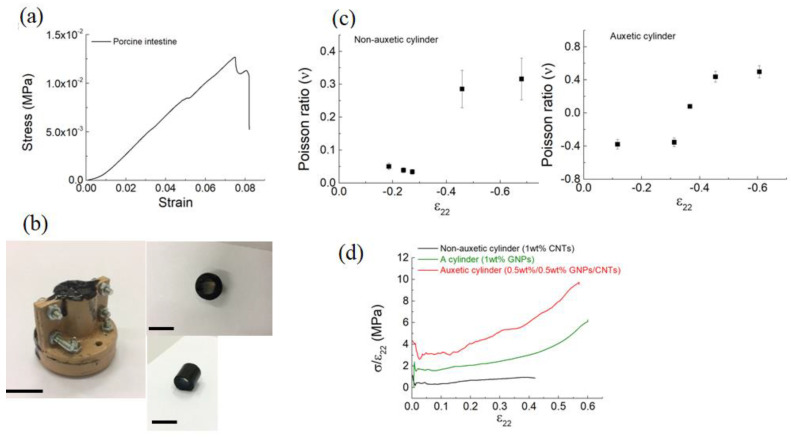
(**a**) Load–strain curve of the porcine intestine. (**b**) Digital pictures (side view) of the auxetic cylinder (i.e., 0.5 wt%/0.5 wt% CNTs/GNPs). The scale bars indicate 10 mm. (**c**) Measured Poisson’s ratio versus compressive strain for the non-auxetic and auxetic cylinders. (**d**) Measured secant modulus as a function of the strain of the non-auxetic (i.e., 1 wt% CNTs) and auxetic (i.e., 1 wt% GNPs and 0.5 wt%/0.5 wt% CNTs/GNPs) cylinders. All the strains are engineering strains.

In Figure 4a, the stress–strain curve of the porcine intestine is reported; from this curve, we obtained the data of elastic modulus (0.1 MPa) and tensile strength (1.25 × 10^−2^ MPa) that were in agreement with those reported by Barducci et al. [22]. Moreover, Figure 4b shows the mold used to prepare the samples and the image of the obtained hollow cylinder. 

The Poisson’s ratio (Figure 4c) and the secant modulus (Figure 4d) were both tested as crucial for the composites’s properties. As shown in Figure 4c, the non-auxetic cylinder Poisson’s ratios varied between +0.05 and +0.31 respectively, when a small and high compressive strain was applied. Comparatively, lateral shrinkage rather than expansion was observed for the auxetic cylinder (Figure 4c), showing a minimum Poisson ratio of ~−0.4. Under compression (Figure 4d), the non-auxetic cylinder showed a classical linear plateau behavior. Alternatively, the auxetic cylinder did not exhibit any plateau but an extended linear elasticity or resilience region. These results are in keeping with the simulation studies reported in ref. [23] where the engineering constants (Young’s modulus and Poisson’s ratio) of auxetic composites consisting of cylindrical materials influenced the effective mechanical properties of the whole composite.

The rule of mixture predicts the synergy between different—assumed immiscible—phases. This model can thus be applied to predict the effect that was observed experimentally. For a composite formed by two immiscible inclusions and assuming isostrain condition, the mechanical strength of the composite is defined by using the rule mixture:

*σ* ≈ *σ_m_* (1 − *f*_1_ − *f*_2_) + *σ*^1^*f*_1_ + *σ*^2^*f*_2_ (7)

where $\sigma_{m}$ is the mechanical resistance of the matrix, *σ* is the mechanical resistance of the single phase, and *f*_1_ and *f*_2_ are the volume percentages of each single phase. Being *σ* $\gg \sigma_{m}\;$, the mechanical properties of the composite increase.

X-ray microtomography is a non-destructive analysis technique that allows visualizing and quantifying samples’ internal structure by generating three-dimensional digital maps with a high resolution (down to the submicron level) [24,25]. In the specific, the result of the microtomographic investigation is a three-dimensional grey-level image proportional to the X-ray attenuation coefficient of the sample (which, for the same energy, is a function of the density and the atomic number of the exciting material), which allows the observation and measurement of the properties of objects (e.g., shape, size, distribution and orientation of fractures, pores, crystals, etc.), entirely avoiding the stereological corrections needed for measurements carried out in two dimensions [26].

**Figure 5:**
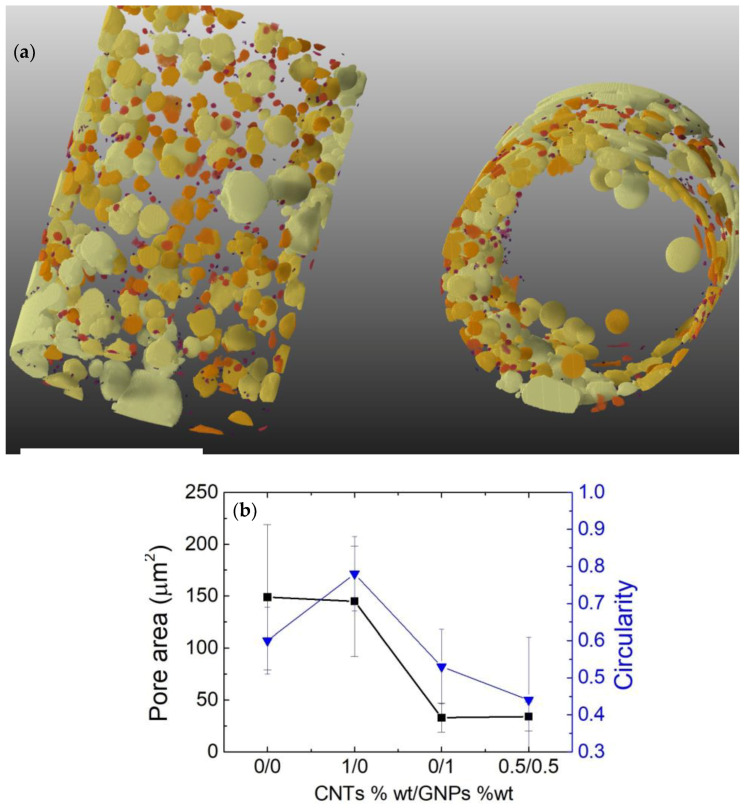
(**a**) Snapshots of μ-CT analysis showing the volume distribution of the bubbles around the hollow middle cylinder. The scale bars indicate 10 mm. (**b**) Pore area and circularity data of the pores as a function of the CNTs/GNPs weight ratio.

Figure 5a shows the results of the μ-CT analysis of the auxetic cylinder. The raw data acquired by the X-ray microscope were processed using the tomographic reconstruction, producing 967 cross-sectional, grey-scale digital images. The cylindrical sample was found to be 10 mm in diameter and 20 mm in height.

To build the composites, a fermentation-assisted method was used. Accordingly, a reaction state was set within a solution containing yeast and sugar, the released CO_2_ bubbles were trapped by the cross-linking agent in the SR at increasing viscosity with the polymerization degree [11]. As soon as gelation started, the accumulation of escaping CO_2_ generated pores, resulting in the creation of a porous cylinder. In the specific, bubbles set aggregated on the external surface of the composited cylinder. It has to be highlighted that collapsed circular pores were obtained by the composition 0.5 wt%/0.5 wt% CNTs/GNPs. Thus, the bubble radius deformation is to be considered directly proportional to the stress in the coating times its thickness (“interfacial tension”). The interfacial tension is thus the main responsible for the bubble deformation and for the final collapsed structure once gelling occurs. Moreover, according to the results presented in reference [27], the stress of the GNP film was found to be higher than the CNTs one; the finding took into account the observed collapsed structures in the SR/CNTs/GNPs composites.

Pore sizes and shapes were analyzed as well as the statistics from the cross-sectional analysis of the auxetic cylinder (Figure 5b). When considering the circularity (measure of how circular each particle is), particles were modelled as ellipses. To mention, a particlewith a circularity of 0 is a straight line, while a perfect circle carries a circularity of 1.

According to the derived data, we demonstrated that in the auxetic cylinder, the pores were not circular, which could be explained by our previously proposed model [11], i.e., the assembly of graphene/nanotubes at a liquid–liquid immiscible interface (water and silicone in this case) is driven by the interfacial tension of the bubble shell produced during the fermentation.

**Figure 6:**
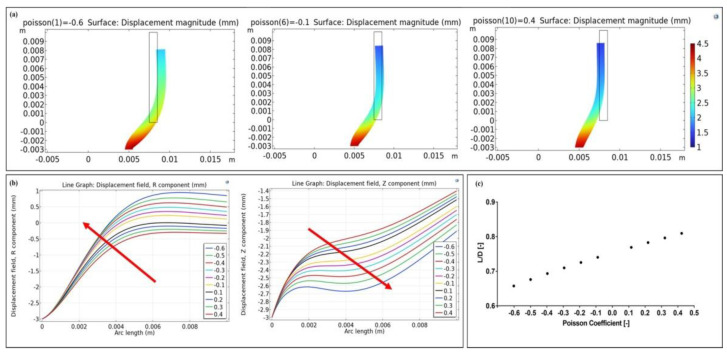
Results of the FE models. (**a**) The frame shows the magnitude of the silicone tube when three different Poisson coefficients were simulated (i.e., −0.6, −0.1, 0.4). (**b**) Displacement along r and z of the inner wall of the silicone tube (blue line, Figure 1c) as the Poisson coefficient varied. The red arrows indicate the increase of the auxetic feature. (**c**) The *L*/*D* ratio as the Poison coefficient varied, showing that the auxetic behavior decreased the pressure loss in the tube.

An FE model was implemented to validate the use of the auxetic silicone tubes with different Poisson’s coefficients as intestinal lengtheners (Figure 6a). Figure 6a shows the deformation of the structures (scale factor = 1) along with the magnitude displacement. As shown in Figure 6b, the tube displacement along r increased with the Poisson coefficient, whereas the displacement along z decreased. Consequently, the tube’s final diameter (*D*) increased with the auxetic behavior, whereas its final length (*L*) decreased, and the ratio *L*/*D* decreased with the auxetic behavior (Figure 6c). This suggested that a lower pressure loss was achieved in the most auxetic tube (ν = −0.6) compared to the non-auxetic one (ν = 0.4), since *L*/*D* is directly proportional to the global coefficient of friction (*k*), as in Equation (7), where f is the Fanning friction factor [28]:

(8) $$k=4f\frac{L}{D}$$

Our findings suggest that the reduction of pressure loss linked to the auxetic tube will synergize with the LILT anatomical changes: in LILT, as per our mathematical analysis, the critical result was that the volume is reduced by a factor of two. At the same time, the total surface area (SA) remained unchanged, and the length doubled. Because the bowel was halved, but the SA remained the same, LILT is increasing the SA/V by two. The latter, ultimately, will maximize the exposure of the bowel content and nutrients to the mucosal surface, thus improving the absorptive efficiency. At the same time, the connection with an artificial auxetic intestine will reduce the afterload for chyme transfer. These results are encouraging in view of a recent study [29] showing that a treatment with the yeast-enriched beer favored the reduction of pro-inflammatory molecules, contributing to an increase in the concentration of anti-inflammatory cytokines. The beneficial effects of a yeast-enriched beer on gut microbiota modulation will pave the way for a safe utilization of such materials in the LILT procedure.

## 4. Conclusions

The contribution of this work is the realization of a design and model matching the mechanical properties of auxetic and non-auxetic materials with periodic Poisson’s ratio variation. A biogenic method to transform non-auxetic silicone rubber to auxetic rubber was reported, along with a demonstration of its crucial role in the mechanical design of soft hybrid auxetic materials. By showing that such materials mimic the intestinal peristalsis, these findings encourage their utilization in a challenging environment like that of the intestine.

## Figures and Tables

**Table 1:** Properties of the involved structures.

	Silicone Cylinder	Intestine
Young’s modulus [MPa]	1.8	0.1
Density [Kg m^−3^]	1000	1000
Poisson Coefficient (ν) [-]	From −0.6 to 0.4 with 0.1 step (Poisson Coefficient equal to 0 was not considered)	0.49 [21]

## Data Availability

The data presented in this study are available on request from the corresponding author.
